# Characterization of Photochromic Dye Solar Cells Using Small-Signal Perturbation Techniques

**DOI:** 10.1021/acsaem.1c01204

**Published:** 2021-08-04

**Authors:** Antonio J. Riquelme, Valid Mwatati Mwalukuku, Patricia Sánchez-Fernández, Johan Liotier, Renán Escalante, Gerko Oskam, Renaud Demadrille, Juan A. Anta

**Affiliations:** † Área de Química Física, Universidad Pablo de Olavide, E-41013 Seville, Spain; ‡ University Grenoble Alpes, CEA, CNRS, Interdisciplinary Research Institute of Grenoble (IRIG), Molecular Systems and NanoMaterials for Energy and Health (SyMMES), F-38000 Grenoble, France; § Department of Applied Physics, CINVESTAV-IPN, Mérida, Yucatán 97310, Mexico

**Keywords:** photochromic, electrochemistry, impedance, dye-sensitized solar cell, IMPS, small-signal perturbation

## Abstract

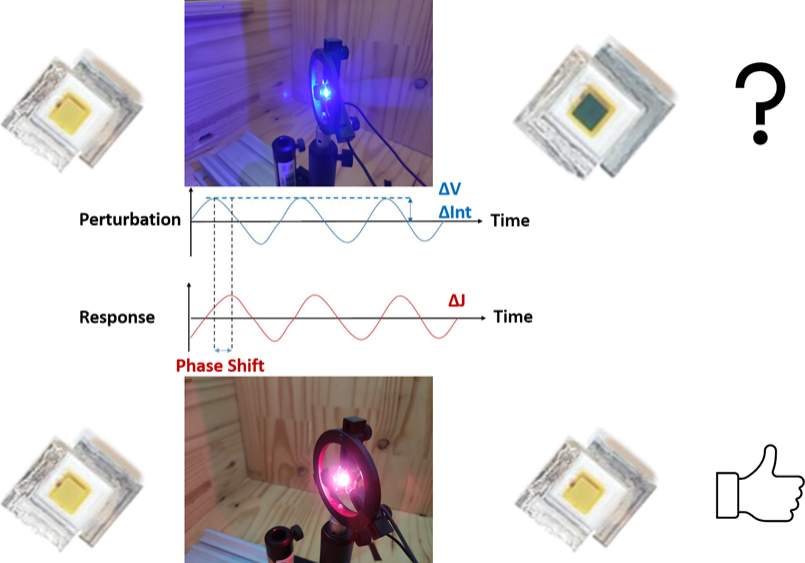

Photochromic dye-sensitized solar cells (DSSCs) are novel semi-transparent photovoltaic devices that self-adjust their optical properties to the irradiation conditions, a feature that makes them especially suitable for building integrated photovoltaics. These novel solar cells have already achieved efficiencies above 4%, and there are multiple pathways to improve the performance. In this work, we conduct a full characterization of DSSCs with the photochromic dye NPI, combining electrical impedance spectroscopy (EIS) and intensity-modulated photocurrent spectroscopy (IMPS). We argue that the inherent properties of the photochromic dye, which result in a modification of the functioning of the solar cell by the optical excitation that also acts as a probe, pose unique challenges to the interpretation of the results using conventional models. Absorption of light in the visible range significantly increases when the NPI dye is in the activated state; however, the recombination rate also increases, thus limiting the efficiency. We identify and quantify the mechanism of enhanced recombination when the photochromic dye is activated using a combination of EIS and IMPS. From the comparison to a state-of-the-art reference dye (RK1), we were able to detect a new feature in the IMPS spectrum that is associated with the optical activation of the photochromic dye, providing a useful tool for assessing the electronic behavior of the device under different conditions of light excitation. This study provides guidelines to adequate characterization protocols of photochromic solar cells and essential insights on the interfacial electronic processes.

## Introduction

Dye-sensitized solar cells (DSSCs) are an emerging photovoltaic technology that has initiated its industrial development.^1^ These cells reach high efficiencies of up to 14% on a laboratory scale,^2−4^ excellent performance upon scaling to small modules,^5−7^ and a remarkable stability.^8−10^ The low cost of the raw materials^11^ compared to other technologies and the simplicity and small environmental impact of their manufacturing make this technology suitable for large-scale production. All these features, together with the possibility to fabricate semitransparent and colorful^6^ solar cells, make them appealing for mass market applications such as in building-integrated photovoltaics (BIPV).^12^ Recently, we reported a breakthrough concept on photochromic DSSCs^13^ that simultaneously self-adapt their level of transparency and photovoltaic performance with sunlight intensity; an efficiency of more than 4% was achieved with the activated photochromic solar cells.

**Figure 1:**
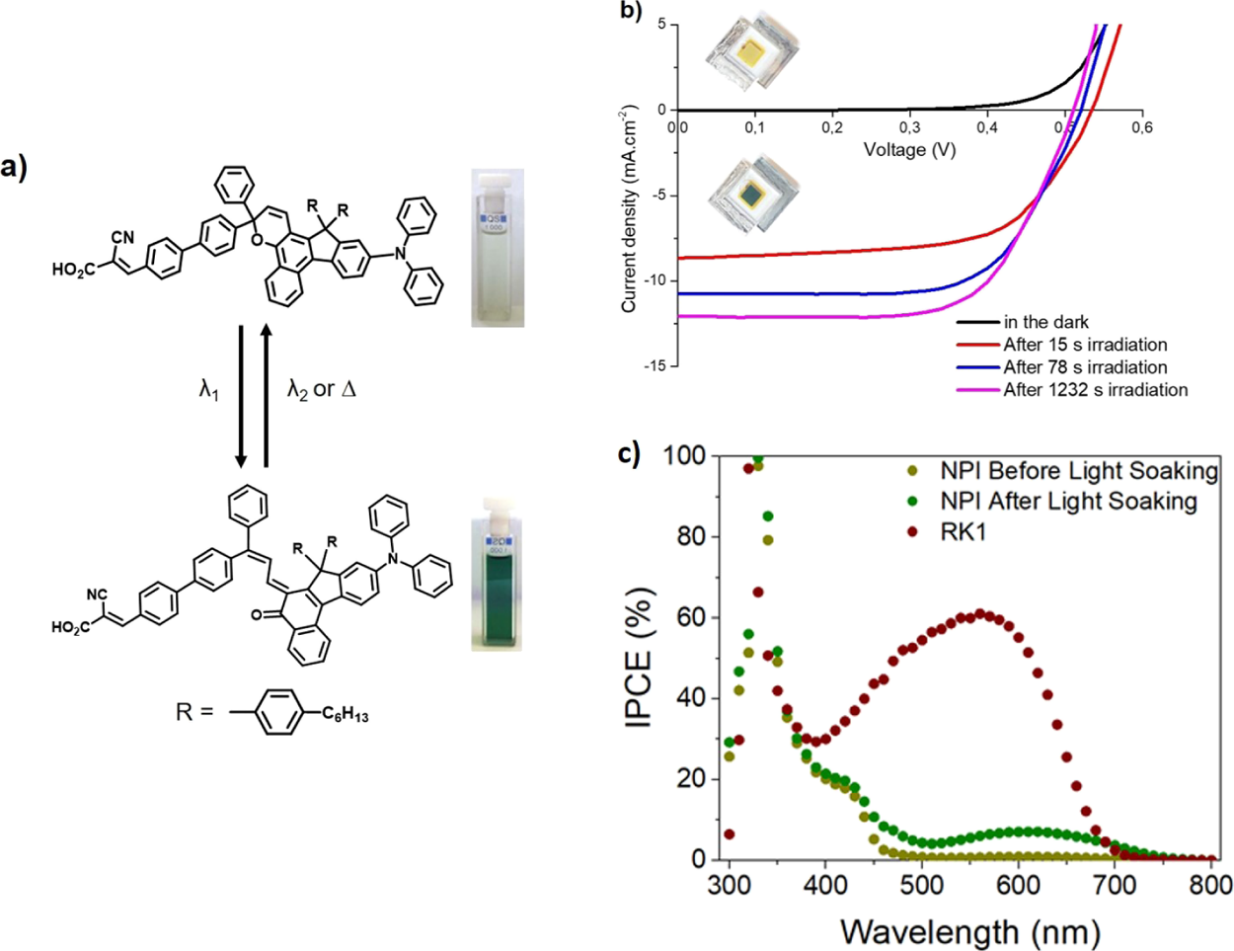
(a) Interconversion process of the NPI photochromic dye (with a 2,2-diphenyl-2*H*-indeno[2,1*f*]naphtho[1,2-*b*] pyran core) where the uncolored, closed form isomer is activated by ultraviolet light absorption generating the opened, colored isomers and the reverse process is achieved thermally. (b) Current–voltage response of DSSCs based on photochromic NPI with the yellow cell under dark conditions and the green cell at the photostationary state following irradiation. (c) Incident photon-to-electron conversion efficiency (IPCE) of a non-photochromic RK1 DSSC and a photochromic NPI before and after reaching the photostationary state.

Our best photochromic dye, NPI, switches from light yellow to a dark green hue under irradiation, related to a change from the closed to an open configuration, respectively,^13^ as shown in Figure 1. The effect of light soaking is observed not only in the short-circuit photocurrent but also in the incident photon-to-electron conversion efficiency (IPCE), which changes significantly in the 500–700 nm region, in contrast to a non-photochromic dye such as RK1. This color change happens quickly, related to the fast activation kinetics upon irradiation within the absorption range of the closed-form isomer of the dye. However, the opposite process is much slower, as it takes several hours before the photochromic dye reaches the fully deactivated state.^13^ To tackle the challenge of developing new photochromic materials with higher efficiencies and faster self-adjustable optical properties, a better understanding of the interfacial processes, as well as the recombination and transport properties of the devices is needed. In this regard, a thorough fundamental study and the development of a reliable measurement procedure that takes into account the changing behavior of the dye under illumination are required.

Small-signal perturbation optoelectronic techniques have been widely used to separate the processes occurring at different time scales in the device. The main technique to obtain information on transport and recombination processes is electrochemical impedance spectroscopy (EIS),^14−21^ where a small voltage modulation is superimposed on the steady-state open-circuit voltage, and the modulated current is measured. On the other hand, combined with EIS, intensity-modulated photocurrent spectroscopy (IMPS),^22−33^ where a small light intensity modulation is superimposed on a constant illumination intensity, is a powerful tool to study and separate transport and recombination processes. Therefore, complementary to this EIS study, the IMPS^30,34^ technique has been applied to these devices.

In order to interpret EIS measurements, spectra are generally fit to an equivalent circuit, and information is extracted from the circuit elements. Generally, the equivalent circuit used to analyze DSSCs was proposed and developed by Bisquert and co-authors,^20,35^ as shown in Figure S2. EIS has proven to be a remarkably powerful tool to understand and quantify transport and recombination processes. However, depending on the system under study, complications may arise, for example, the time constants of different processes may be too close to separate and, for some cases, the system may not be stable under a voltage modulation. On the other hand, IMPS is generally performed under short-circuit conditions; the results can be interpreted as a frequency-dependent external quantum efficiency (EQE), where the zero frequency limit of the transfer function generally reproduces the steady-state EQE.^36,37^ Therefore, where EIS gives information on voltage-dependent processes, IMPS provides separate information on charge transport.

The particularities of photochromic dyes may result in a change of the system during the measurement itself, because the illumination used to define a specific quasi-Fermi level in the TiO_2_ film or even the modulated light intensity used in IMPS could also activate the dye molecules. This in turn, may change the charge stored and the kinetics of the transport and recombination processes as a function of voltage and light intensity. Therefore, a clear and simple procedure to measure and analyze the response of this novel type of photochromic devices is needed. In this work, we focus on the peculiarities of the small-signal perturbation analysis of a photochromic dye-sensitized solar cell in comparison with a conventional DSSC, and we show that the activation of the photochromic dye accelerates recombination without shifting the band edges of the oxide. With the goal to unravel how the photochromic properties of the dye influence the photovoltaic processes of DSSCs, to our knowledge, this work provides the first thorough study combining EIS and IMPS to assess the effect of voltage and light intensity modulations on the determination of the transport and recombination characteristics in photochromic devices. Furthermore, we intend to cast light on the origin of the decrease of the photovoltage observed in DSSCs prepared with the NPI photochromic dye when the cell is optically activated.

## Results and Discussion

In 2020,^13^ we reported a new photochromic naphtpyran-based dye that switches color upon irradiation from pale yellow to dark green. In order to enhance the transparency of the device, a 13 μm mesoporous TiO_2_ layer was used as the working electrode, while a platinum-coated FTO served as the counter electrode. To avoid any regeneration limitation of the device, a non-viscous, iodine-based electrolyte, optimized specifically for the NPI dye was used. In our previous work, we also reported that when photochromic dyes are activated upon illumination, a stunning rise of the photocurrent occurs until a photostationary state is reached. However, the activated state also induces a decrease of the open-circuit voltage, as can be observed in Figure 1. A priori, this behavior could be explained by either a shift in the bands of the metal oxide semiconductor or by an acceleration of the recombination kinetics. Therefore, it is very important to clarify whether this behavior is intrinsic to the new dye material or is related to the activation process. Our first study highlighted, with help of theoretical and experimental data, that the acceleration of the recombination process is the main reason behind this observation. In order to better understand all the processes that may lead to this behavior, a full small-signal perturbation study has been conducted on the photochromic dye solar cell, both for the activated and the deactivated state. For the sake of comparison, the same study was conducted on the well known, fully organic, non-photochromic, and commercially available RK1 dye,^8^ whose current–voltage curve is given in Figure S1.

**Figure 2:**
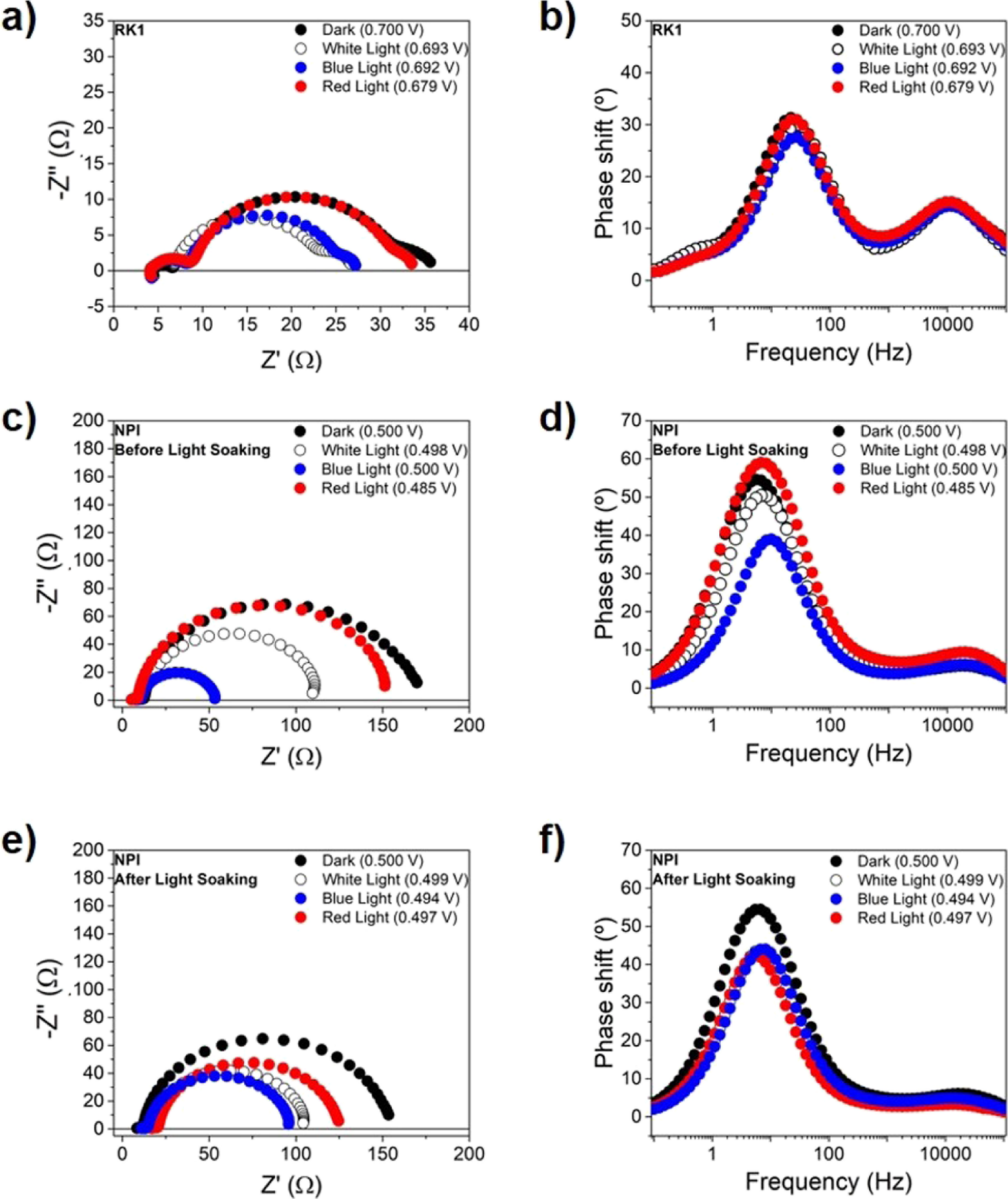
EIS results under monochromatic LED illumination for an RK1 solar cell (a,b) and an NPI solar cell, both before (c,d) and after a (e,f) light soaking treatment. The measurements were performed at an applied voltage equal to the open-circuit voltage that is obtained under white light 1 sun illumination using a solar simulator; about 0.7 V for the RK1 cell and 0.5 V for the NPI cell. The Nyquist plots are shown on the left (a,c,e), and the Bode plots are shown on the right (b,d,f).

As can be observed in panels a and b in Figure 2, the EIS results show the typical three arcs for the RK1 dye solar cells, with the equivalent peaks in the Bode plot.^20^ These three signals appear at 10–50 kHz and 5–500 and 0.1–1 Hz, respectively. The first, high-frequency semicircle can be related to the platinum counter electrode,^38^ as shown in Figure S3. The high-frequency feature disappears by changing the counter electrode material from platinum to PEDOT. The feature at low frequencies, on the other hand, is associated with electrolyte diffusion.^39,40^ These two signals are of little interest for the focus of this study; therefore, we limit ourselves to the discussion of the main arc of the spectra in the 5–500 Hz range, linked to the recombination processes in the DSSC.^20^ As a simplification, the size of the real part of this arc is approximately equal to the recombination resistance. In well-performing dye solar cells, the recombination process corresponds to electron transfer from TiO_2_ to tri-iodide in the electrolyte solution. Hence, the recombination resistance is a charge transfer resistance that can also be measured in the dark, upon applying a voltage similar to the open-circuit voltage under illumination, following the superposition principle approximation. We observe a decrease in the recombination resistance for the cell under illumination as compared to the charge transfer resistance in the dark at the same voltage, which is typical for DSSCs and is related to the local increase of the tri-iodide concentration due to the dye regeneration process, thus accelerating recombination.^16,41^ In accordance to the RK1 absorbance spectrum in Figure S5, the absorption coefficient for the red light is almost zero, which translates to a significantly lower recombination current at V_oc_ and, thus, a lower triiodide generation rate; hence, the recombination behavior is very similar to the cell under dark conditions. In the Bode plot in Figure 2b, no shift in the frequency of the apex of the recombination arc is observed, illustrating that the wavelength of the illumination does not affect the recombination rate.

The same experiments were carried out with NPI solar cells and the results are presented in panels (c–f) in Figure 2. Before light soaking, the general trends are the same as for the RK1 solar cell; however, the decrease of the recombination resistance is clearly more dramatic upon illumination, especially for the white and blue illumination, which is related to the properties of the photochromic dye. This effect is also observed when comparing the activated and deactivated states of the cell, especially for the EIS measurements under dark conditions and with red illumination. As for the blue and white light measurements, it is important to mention that, even before light soaking, a substantial amount of dye molecules get activated during the small perturbation measurement itself (see Figure S6). This makes it difficult to compare the activated and non-activated states of the cells for light excitation where the dye absorbs. This is clearly seen in Figure 2d, where the impedance peak for blue light gets displaced toward higher frequencies with respect to the dark and red illumination.

**Figure 3:**
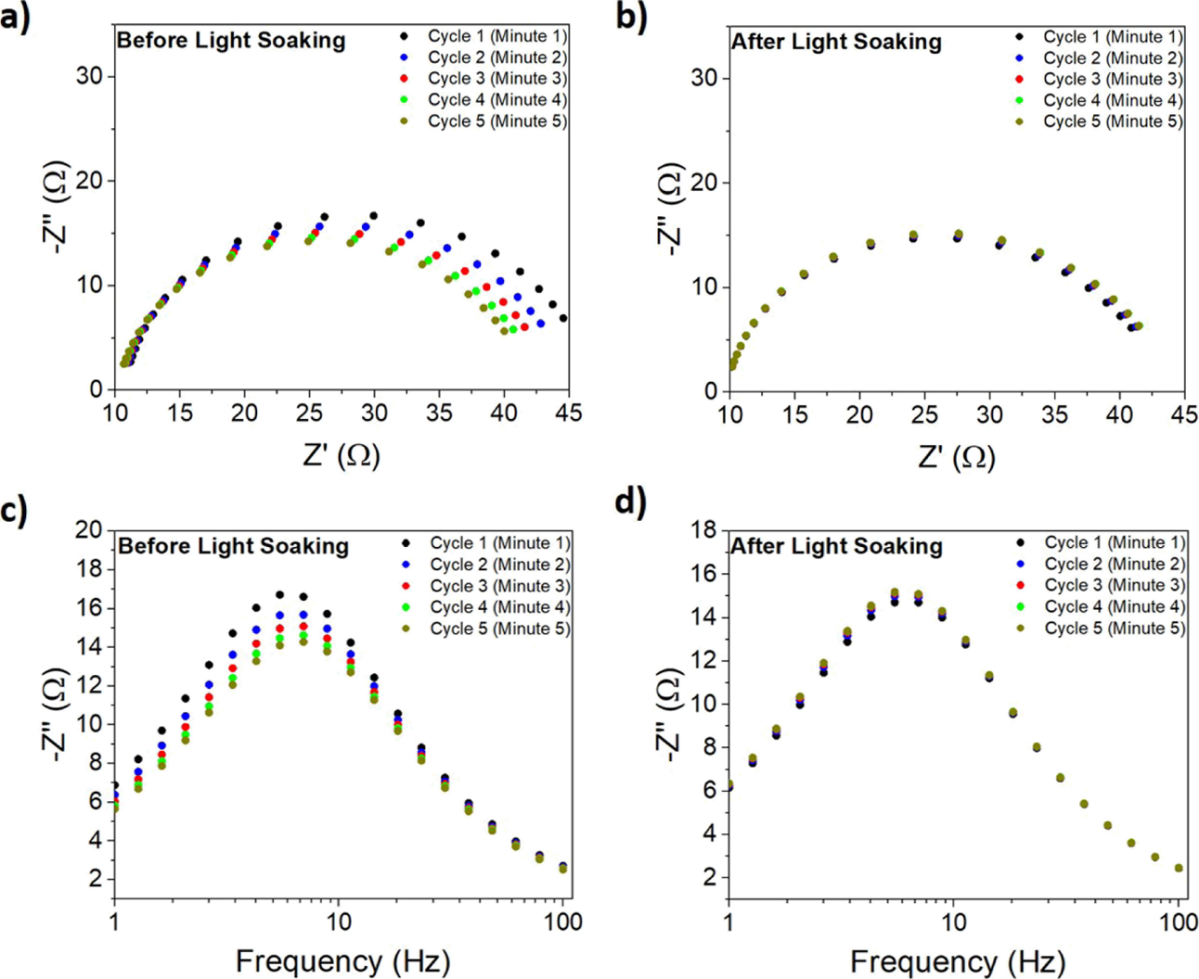
Nyquist and Cole–Cole plots of impedance measurements under blue light illumination before (a,c) and after (b,d) light soaking in the 1–100 Hz frequency range.

To confirm this interpretation, we have run a very quick (5 min in total) impedance experiment under blue light illumination restricted to the 1–100 Hz frequency range (where the recombination arc appears). The results are shown in Figure 3: The observed acceleration of the recombination rate illustrates that the cell is quickly activated. This is displayed as a decrease of the semicircle diameter, hence the recombination resistance, in the Nyquist plot and a shift of the peak toward higher frequencies (Figure 3c,d), corresponding to an increase in the recombination rate constant, both in the time span of a few minutes.

**Figure 4:**
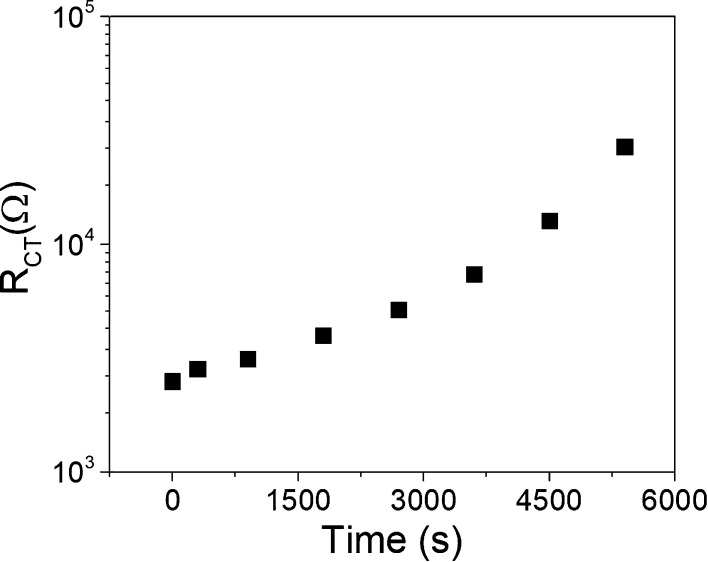
Charge transfer resistance from the EIS experiment under dark conditions at 0.35 V external voltage applied to an NPI dye-sensitized solar cell as a function of time after 15 min of light soaking following the measurement procedure described in the Experimental Section.

The impact on the charge transfer resistance of the deactivation process can be observed in Figure 4 as a function of time in the dark after light soaking. The increase in the charge transfer resistance under dark conditions with the deactivation process of the molecule is obvious and can be compared to Figure S7, where we observe that most of the deactivation of NPI in a full cell takes place within the first 4 h. This result also indicates that the discoloration process in a photochromic dye based cell slows down the recombination process, or in other words, the activation of the dye accelerates recombination.

From the Bode plots in Figure 2 panels (d,f), it can be observed that unlike in panel a, the main signal frequency peak shifts somewhat to higher frequencies upon dye activation, indicating that either the activation of the photochromic dye induces a faster recombination process or produces a band shift. It is also noticeable that the low-frequency feature observed with RK1 disappears for NPI, which can be explained by the fact that an optimized electrolyte was used for the NPI devices, as described in the Experimental Section.

The EIS results, as shown in Figure 2, can be fitted to an equivalent circuit in order to better analyze the behavior of the cells under dark conditions and with illumination. Since it was not possible to get an accurate fit using the classical Bisquert and co-workers equivalent circuit,^20,35^ the main arc in the 5–500 Hz range related to the charge transfer/recombination process was fitted to a parallel -RC- element, where the resistance is considered to be the charge transfer (dark) or recombination resistance of the device (*R*_CT_), while the capacitance is the chemical capacitance (*C*_μ_). As a specific characteristic of DSSCs, both parameters generally show an exponential voltage dependence^42^
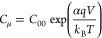
1where *C*_00_ is a constant, α is the trap distribution parameter, *q* is the elemental charge, *V* is the voltage applied to obtain each impedance, *k*_B_ is the Boltzmann constant, and *T* is the temperature.
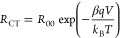
2where *R*_00_ is a constant and β is the transfer parameter or recombination parameter.

**Figure 5:**
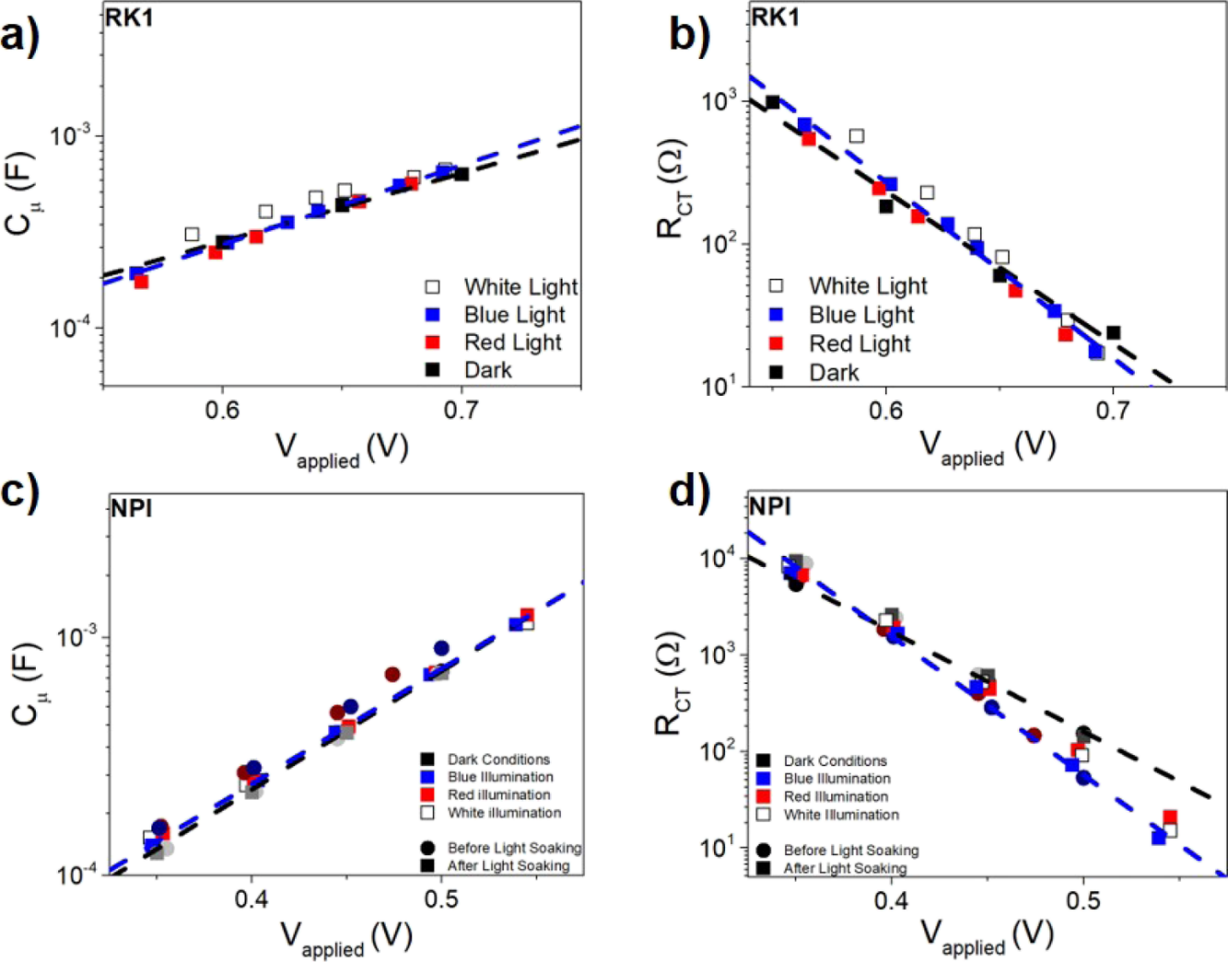
Capacitance (a,c) and resistance (b,d) vs applied voltage for RK1 (a,b) and NPI (c,d) solar cells, in the dark and under LED monochromatic illumination. The results were obtained from fitting the main arcs in Figure 2 to a parallel -RC- circuit. Fits to eqs 1 and 2 are included for the results obtained in the dark and under blue LED illumination in order to highlight the general trends observed. Note that the applied voltage under illumination corresponds to the open-circuit voltage obtained at the specific intensity.

Figure 5 shows that the resistance and capacitance of the charge transfer/recombination signal of the EIS have an exponential voltage dependence as described in eqs 1 and 2. For both the RK1 and NPI solar cells, the chemical capacitance is independent of the illumination conditions, indicating that the band edges do not shift. The same result is found for the NPI-based cell in the activated and deactivated states illustrating that the activation process induces no band shift,^19,43^ as was previously indicated by DFT calculations.^13^ Thus, we can conclude that the shift toward higher frequencies of the main signal in Figure 2 panels (d,f) is as a result of an increased recombination rate. Therefore, the decrease of the *V*_oc_ associated with the activation of the dye is due to kinetic reasons only.^43,44^ However, as shown in Figure S4, there is a band shift between the NPI and RK1 dyes of around 0.15 V. Looking at the values obtained for the recombination resistance, as the light intensity increases and the open-circuit voltage approaches the values obtained at 1 sun under the solar simulator and, hence, under practical operating conditions, differences between the open and closed forms of the dye become more apparent. This was already observed in our previous study^13^ and in Figure 4.

At this point, it is important to remember that, by comparing at the same applied/generated potential, we ensure that the electron quasi-Fermi level and the electron density in the photoanode for the experiments carried out with different illumination sources is the same.^20,45,46^ In this respect, the results of the chemical capacitance and the recombination resistance in Figure 5a–c are quite conclusive. In spite of using different illumination conditions, the chemical capacitance is the same at the same value of the applied voltage. Furthermore, the recombination resistance is the same for the RK1 reference cell. This proves that photochromism is the only factor affecting the observed increase/decrease of the recombination resistance.

**Figure 6:**
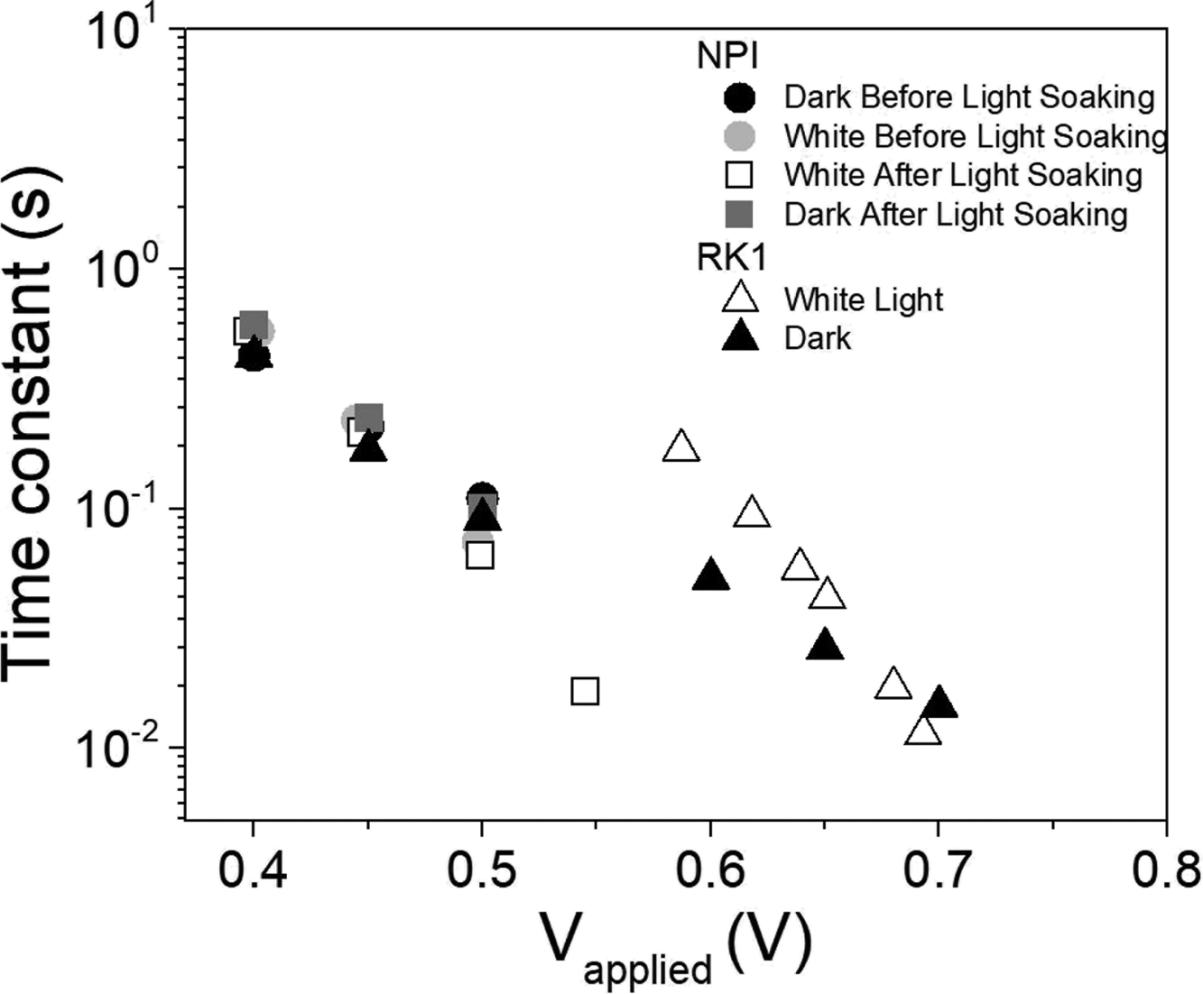
Time constants associated to the recombination feature in the Bode plots in panels (b,d,f) of Figure 2 under dark conditions and under white light illumination for the RK1 DSSC, and the activated and deactivated NPI-based solar cell.

The α and β parameters included in Tables S1 and S2 are within the range normally reported for DSSCs (0.15–0.35 for α and 0.5–0.8 for β).^19,47−49^ Although there is an increase in the β values upon activation of the photochromic dye, this change is not sufficient to imply a change in the dominant recombination pathway corresponding to triiodide reduction. In addition to the α and β values obtained from fitting of the EIS to the parallel -RC- element, the time constants associated to this recombination feature, as shown in Figures 6 and S8, are also an important source of information.

The time constants observed in Figure 6 correspond to the effective electron lifetime,^50^ which decreases in a dramatic way as both the illumination intensity and the open-circuit voltage increase. If we compare this behavior to the trend shown by the recombination resistance in Figure 5, it is easy to understand that as the recombination resistance decreases and hence the recombination rate increases, the charge carriers recombine at a higher rate; therefore, their lifetime decreases. It is remarkable that the lifetime versus voltage plots for both RK1 and NPI devices follow the same slope for all the excitation wavelengths as can be observed in Figure S8. This is consistent with the similar α and β values observed in Tables S1 and S2 and suggests that the same recombination process takes place in both devices. The only difference between the time constants obtained for the NPI and RK1 dyes is the band shift of 0.15 V observed in Figure S4.

The diffusion length, which can be described as the average distance that an electronic carrier can travel across the active layer before recombining, is a very useful parameter of the cell, because it can be directly related to the amount of charge that can be collected by the cell. However, due to the complex kinetics of the processes taking place inside the cell, it is very hard to obtain an accurate value for this parameter without using complicated random walk models. Since the small-signal perturbation techniques allow us to simplify the behavior of the cell, assuming first-order kinetics for recombination, we can obtain to a good approximation the small-signal perturbation charge carrier diffusion length. This value can be obtained from the EIS^20^ with the following equation
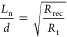
3where *L*_n_ is the small-signal perturbation charge carrier diffusion length, d is the thickness of the semiconductor layer, which is known to be 13 μm, *R*_rec_ is the recombination resistance, and *R*_t_ is the transport resistance.
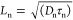
4

However, since it was not possible to fit the EIS to the classical Bisquert and co-workers equivalent circuit^20,35^ because no clear transport line was present in the spectra, eq 3 cannot be applied to get a well-defined diffusion length. However, the expression can still be used to state the qualitative relation between the diffusion length and the recombination resistance.

For these cells, EIS alone failed to provide information about the small-signal perturbation electron diffusion length. Thus, this parameter can alternatively be calculated by eq 4,^24^ where *D*_n_ is the electron diffusion coefficient, which can be obtained from IMPS, and τ_n_ is the electron effective lifetime, as shown in Figure 6, provided that both parameters are measured at the same quasi-Fermi level position.^51−54^

**Figure 7:**
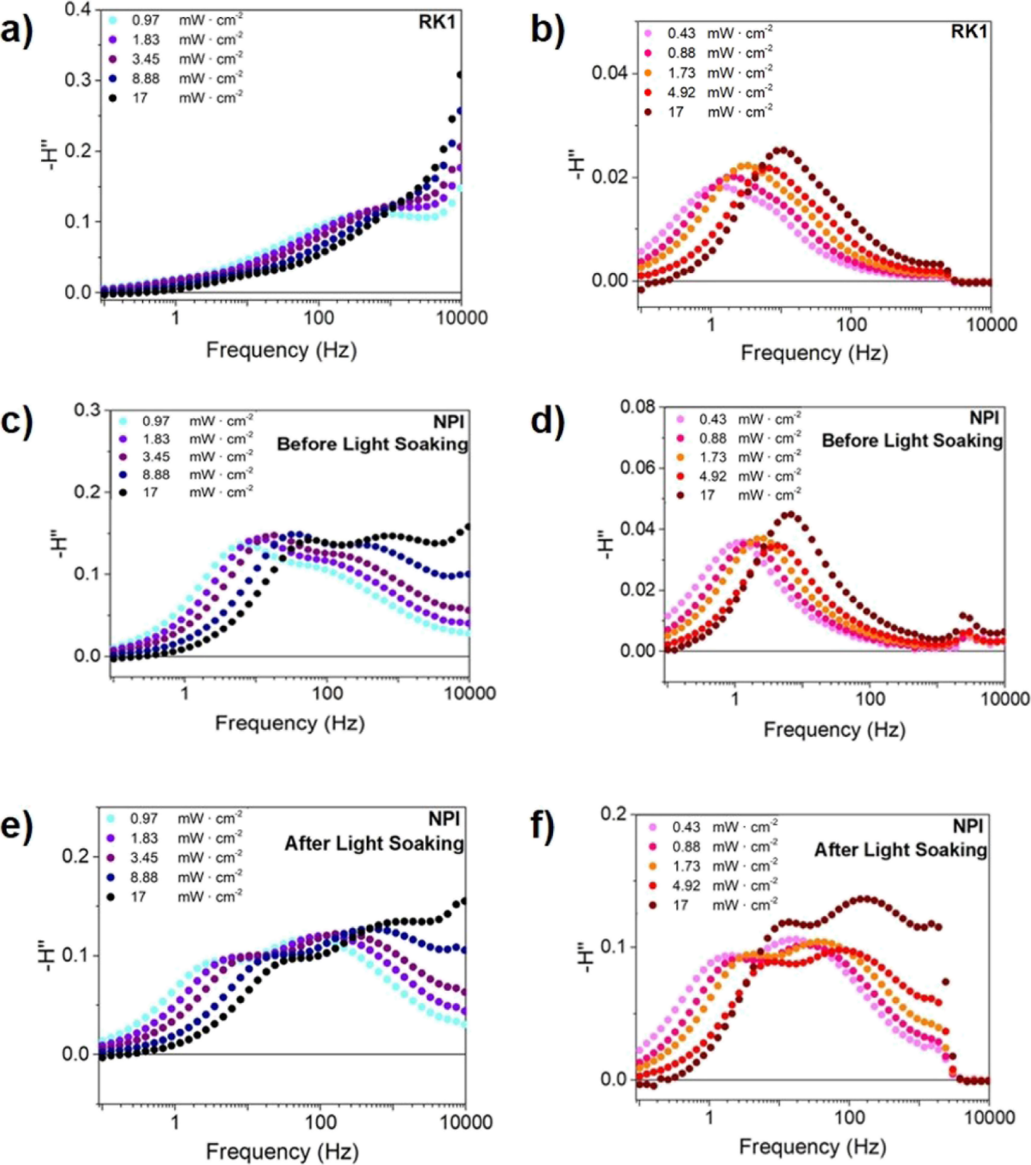
Cole–Cole plots of the IMPS measurements of an RK1 solar cell (a,b) and a photochromic NPI solar cell, both deactivated (c,d) and activated (e,f) under different light intensities with blue (a,c,e) and red (b,d,f) illumination.

From panels a and b in Figures 7 and S9, it can be seen that there are two distinguishable signals with the conventional non-photochromic dye RK1 as previously reported for various DSSCs and other thin-layer photovoltaic devices.^37,42,55−57^ For a film thickness as large as the TiO_2_ layer of these devices (13 μm), the signal observed in the 10–100 Hz range can be related to the electron transport in this nanostructured mesoporous layer. Some differences between the spectra obtained with the two excitation wavelengths are observed, such as the size of the feature that appears at high frequencies and a shift of 1 order of magnitude of the frequency where the signal associated to electron transport in the TiO_2_ layer is observed. However, the general shape and behavior of two identifiable signals is consistent.

When the same IMPS experiment is carried out on deactivated and activated NPI solar cells (panels c–f in Figure 7), an additional signal in the 100–1000 Hz range appears for the activated dye, which is absent in the RK1 or NPI devices before light soaking under red illumination. However, the feature does appear in the IMPS measurement before light soaking under blue illumination. As discussed before, blue light activates the photochromic dyes making it impossible to measure the fully deactivated state. Hence, differences between the activated and the deactivated states of the dye can only be observed using red illumination. Since the additional signal only appears for the activated dye, it seems to be related to the activation process of the molecules. In addition, the 100–1000 Hz range where it appears corresponds to the millisecond time regime, which would be consistent with a fast change of coloration associated to the activation process when embedded in a solar cell. This result indicates that the only way to truly assess the activation and deactivation processes via optoelectronic small perturbation techniques is by exciting at a wavelength which does not induce photochromism.

**Figure 8:**
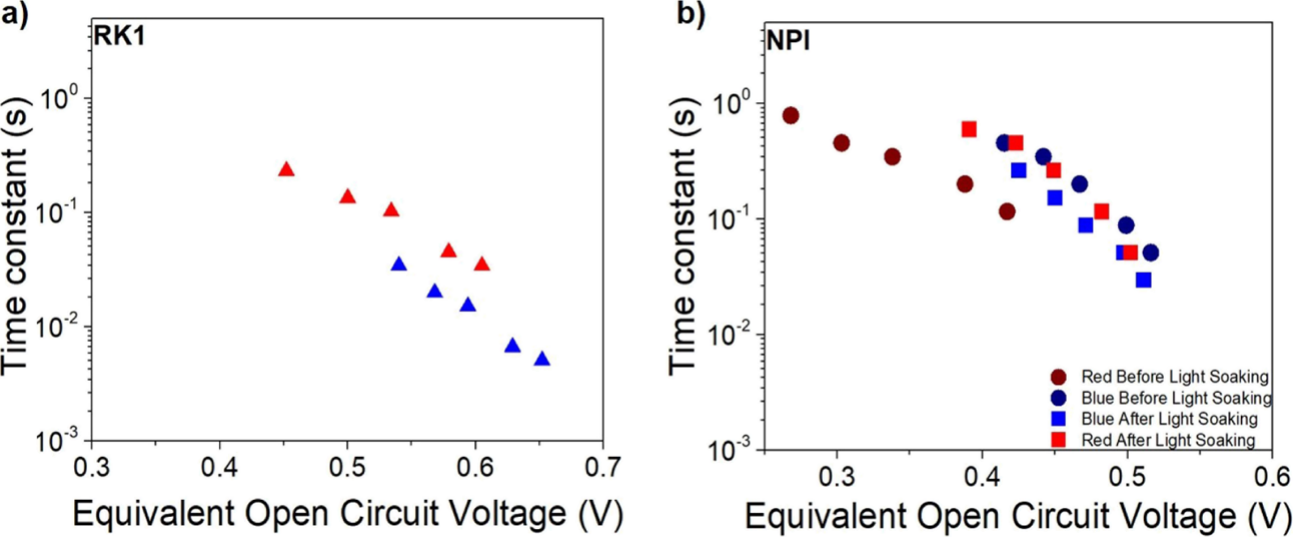
Time constants associated to the main feature of IMPS under red and blue light for an (a) RK1 and (b) NPI device, both before (circles) and after (squares) light soaking obtained from the results shown in Figure 7.

The behavior of the time constants represented in Figure 8 shows an exponential dependence on light intensity that has been ascribed to the transport mechanism of multiple trapping,^19,22,37^ which confirms its origin to be the transport in the mesoporous TiO_2_ layer. As expected, the time constants do not change significantly when the dye is fully activated (blue illumination in both cases and red illumination after light soaking). However, electron transport when the dye is not activated (red illumination before light soaking) appears to be somehow faster. The reason is that since the non-activated dye absorbs much less in the red (Figure S6), the light illumination intensity required to obtain the same value of the equivalent open-circuit voltage is larger. Consequently, the quasi-Fermi level in the TiO_2_ photoanode is higher up in energies and transport becomes faster, in accordance to the multiple trapping model. Knowing this, the small-perturbation electron diffusion coefficient can be obtained by^19^
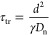
5where τ_tr_ is the time constant for the transport process observed in IMPS, *d* is the thickness of the semiconductor layer, which is known to be 13 μm, and γ is a dimensionless factor that depends on the direction of illumination and the ratio between the absorption length and the thickness of the film, whose value can be approximated to 2.5. Since these values are being used together with the data obtained from EIS at open-circuit voltage, we need to ensure that both parameters are obtained at the same trap occupancy (i.e. at the same position of the Fermi level relative to the conduction band). To do that, a correction, which for DSSCs is known^58^ to be about 0.3 V, is applied (see Figure S10). However, this correction is likely overestimated for the case of the deactivated NPI dye, especially for the red LED IMPS measurements before light soaking due to the low absorbance of the closed dye; at this wavelength, the Fermi level is very close to its position under dark conditions. Since no drift-diffusion models that include photochromic behaviors are currently available, and for the sake of simplicity given that no significant differences in the qualitative behavior under small perturbation techniques have been identified in this study, the same voltage correction has been applied to the NPI dye, with the precaution of only interpreting the higher light intensity values.

By extending the time constant trend line from the EIS measurements to short-circuit conditions (Figure S11) and doing the same with the diffusion coefficients calculated by applying eq 5 to the time constants from IMPS (Figure S12), we obtain a diffusion coefficient of 3 × 10^–11^ m^2^/s for the RK1 and 4 × 10^–12^ – 8 × 10^–11^ m^2^/s for the NPI solar cell. These values are combined using eq 4 to obtain the diffusion lengths (Figure S13) which are found to be 200–500 μm for RK1 and 50–110 μm for NPI. These large values (in comparison with the film thickness) suggest that charge collection in the TiO_2_ approaches 100% in both cases.

**Figure 9:**
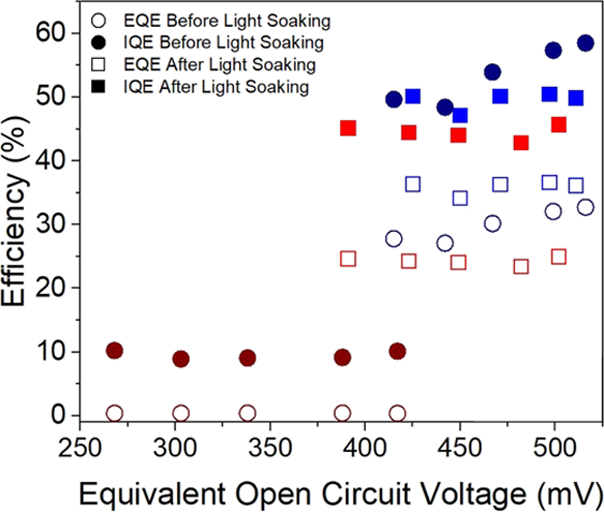
External (hollow symbols) and internal (solid symbols) quantum efficiencies obtained from IMPS measurements under short-circuit conditions for an NPI solar cell in the activated (squares) and deactivated (circles) states under red and blue monochromatic illumination.

In Figure 9, it is noticeable that there are no evident differences between the internal and external quantum efficiencies under blue illumination for the activated and deactivated dye. This is because, as already mentioned, the blue light activates the dye right after starting the measurement, making it impossible to characterize the deactivated state with blue light. The IQE values obtained under red light illumination on the activated cell are similar to those obtained with blue illumination, which is consistent with the diffusion length results. The IQE values of the activated NPI device are similar to those of the RK1 cell, as can be observed in Figure S14, although recombination is faster for the NPI solar cells as compared to the RK1 cells. This could be as a result of regeneration or injection issues in RK1 cell due to limitations of the electrolyte used. The electrolyte used for the NPI cell was a homemade electrolyte specifically optimized for this dye; so, these limitations are avoided. On the other hand, the red illumination on the deactivated cell produced very poor IQE values in apparent contrast with the large diffusion length estimated for the deactivated NPI-based cell under red illumination. It is important to stress, however, that the diffusion length only accounts for the charge collection efficiency; therefore, limitations in the regeneration or poor charge generation due to negligible absorbance can produce low IQE values, while the charge collection efficiency inferred from the diffusion length is high.

## Conclusions

We present a clear and simple procedure to measure and analyze photochromic dye solar cells by optoelectronic small-signal perturbation techniques, distinguishing between the activated and deactivated states of the dye molecule. For this purpose, EIS and IMPS have been used to estimate the effect of the photochromism of these cells on recombination and electronic transport. We have shown that the general behavior of the photochromic NPI dye in an EIS study is fundamentally similar to any other dye previously studied. EIS has been used to determine changes in the recombination kinetics upon activation of the photochromic dye, finding that activation of the molecule leads to a faster recombination process, which induces a lower charge extraction efficiency. This result, combined with the similar electronic diffusion coefficient for RK1 and activated NPI solar cells obtained from IMPS illustrates that there is a decrease in the electronic diffusion length in NPI compared to the reference RK1 dye. However, this effect is not obvious in the IQE results because injection and regeneration issues related to the non-optimized electrolyte limit the RK1 cell performance. This issue was solved for the NPI cells by the use of a homemade electrolyte specifically designed for the NPI dye. Finally, an additional signal characterizing the activated NPI dye with IMPS has been found. Given that this signal is neither present in the deactivated state of the photochromic NPI cells nor in the non-photochromic RK1 cells, these observations led to the conclusion that this signal is related to the activation process of the dye, thus making IMPS an important tool for assessing the activation kinetics of new photochromic dyes developed in the future. We have shown in this work that even when we ensure that the photochromic devices are fully deactivated before the measurement, if this is conducted using short wavelength illumination, the cell is partially activated instantly when starting the measurement, making it impossible to study the deactivated cell. Therefore, if a study on the deactivated dye is aimed at, the use of long-wavelength excitation, which will not activate the photochromic dye, is required.

## Experimental Section

### Fabrication of DSSCs

Transparent photoanodes, commercially available from Solaronix, Switzerland (Ti-Nanoxide HT/SP) with a 13 μm mesoporous TiO_2_ layer and an active area of 0.36 cm^2^, were cleaned using ethanol and treated with a 4.1 mmol L^–1^ TiCl_4_ aqueous suspension at 70 °C for 20 min followed by sintering at 500 °C for 20 min. The previously perforated counter electrodes were coated with a thin layer of platisol (Solaronix, Switzerland) and annealed under air at 500 °C for 20 min. The photoanode sensitization mixture (0.5 mM[dye]/5 mM [CDCA]) coadsorbant dissolved in a 1:1 mixture of CHCl_3_/*t*BuOH for NPI-based cells and 1:1 mixture of CHCl_3_/EtOH for RK1-based cells. Both electrodes were sealed together using 60 μm Surlyn and then filled with the appropriate electrolyte solution, Solaronix HI-30 for RK1 and our optimized homemade one for NPI.^13^ The pre-drilled holes were sealed with a thin glass cover with Surlyn underneath.

### Characterization of the Devices

**Figure 10:**
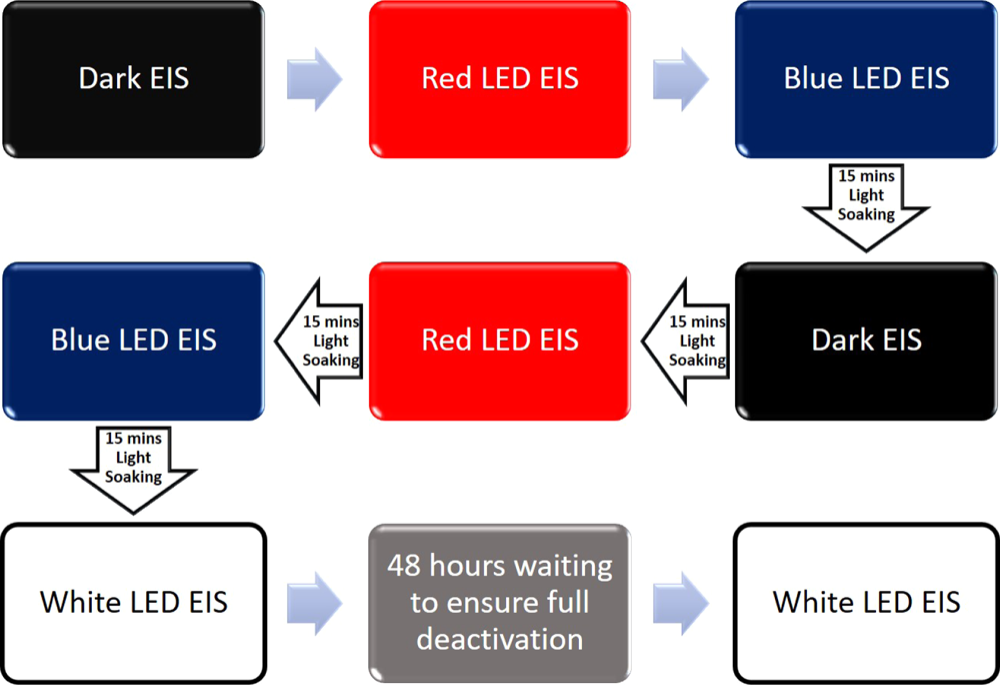
Flow chart of the order in which the EIS measurements were done. Dark EIS after light soaking was performed following the flow chart of Figure S15 to ensure the same level of activation of the dye for all the measurements.

The solar cells were characterized under 1 sun, using an AM1.5G simulator previously calibrated with a reference photodiode (KG-3, Schott). The current–voltage response in the dark and under illumination of the devices were registered by applying an external voltage bias to the devices, and the resulting photocurrent was recorded with a Keithley model 2400 digital source meter (Keithley, USA). A mask was used prior to delimit an illuminated active area of 0.25 cm^2^. The EIS studies were conducted using an Autolab/PGSTAT12/30/302N FRA2 potentiostat. IMPS measurements were carried out by coupling an LED to the PGSTAT302N/FRA32 module by means of an LED communication driver (Autolab). A 0.25 cm^2^ plastic mask was used to limit the area of the cell receiving illumination during the IMPS measurements. EIS measurements under illumination were performed by applying a potential equal to the observed open-circuit voltage under constant illumination by white, blue (480 nm), and red (650 nm) Thorlabs LEDs over a wide range of DC light intensities. A 10 mV perturbation in the 10^–1^ to 10^5^ Hz range was applied. The NOVA 2.1. software was used to generate the data. Z-view equivalent circuit modeling software (Scribner) was used to fit the spectra. A flow chart of the EIS measurements of the NPI cells to ensure that all the different experiments were performed on both the activated and deactivated forms of the dye can be observed in Figure 10.

The EIS measurements under dark conditions were conducted ensuring that no external source of light interfered with the experiment and by applying an external voltage from 0.1 V to the open-circuit voltage obtained under 1 sun illumination.

**Figure 11:**
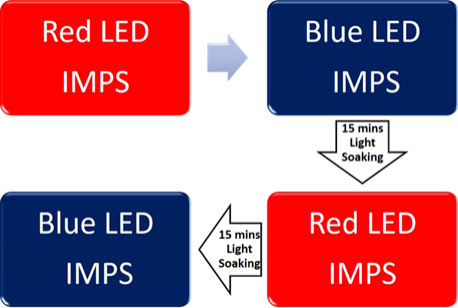
Flow chart of the order in which the IMPS measurements were performed.

The frequency range of the IMPS measurement was restricted to 10^–1^ to 10^4^ Hz due to limitations of the setup (LED and LED driver). The perturbation amplitude was set to 10% of the DC background illumination intensity.^37^ The intensity of the monochromatic light was calibrated using a Thorlabs FDS100 silicon photodiode. IMPS measurements were performed under short-circuit conditions under blue and red illumination using ILH-GD01-SC201 ILS LEDs. The order in which the IMPS measurements were done can be observed in Figure 11.

In this work, we distinguish between the behavior of the solar cells for the deactivated and activated photochromic NPI dye. The change was induced by exposing the cell to the solar simulator (ABET-Sun2000) for 15 min under 100 mW/cm^2^ illumination with an AM 1.5G filter, ensuring that the illuminated area of the solar cell had turned to a dark green color. The light intensity was recorded using a reference monocrystalline silicon solar cell with temperature output (ORIEL, 91150).

**Figure 12:**
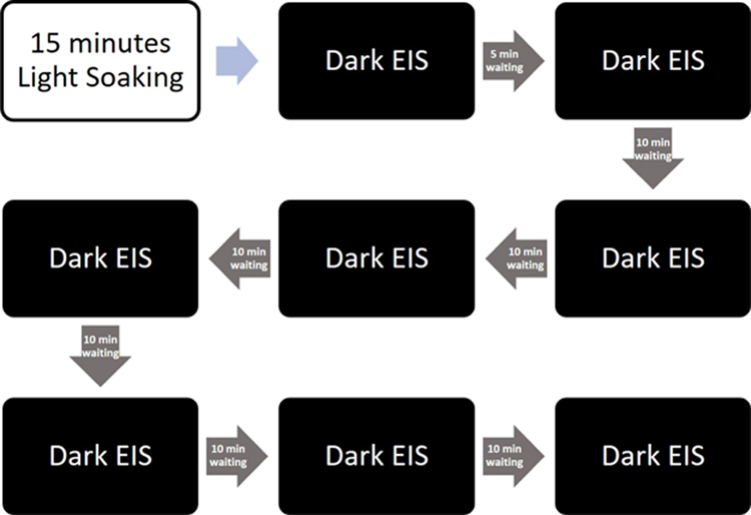
Flow chart of the deactivation kinetics study.

An additional experiment was conducted to study the evolution of the recombination kinetics upon deactivation of the NPI dye to better understand the relation between the activated and deactivated states of the dye and the recombination process. For that purpose, after 15 min of 1 sun illumination, EIS under dark conditions at a fixed 0.35 V external voltage was conducted after different time intervals to study the discoloration process. For more clarity, a flow chart of how this measurements were done can be observed in Figure 12.

In order to include statistical values, two cells of each type of dye were completely characterized by EIS and IMPS.

## Supporting Information Available

Current–voltage curves of the RK1 and NPI dyes, equivalent circuit used to fit the impedance, Bode plots with different counter electrode materials, bandshift under white illumination of the RK1 and NPI dyes, absorbance spectrum of the RK1 and NPI dyes, deactivation kinetics of the NPI dye, tables of the impedance parameters of the RK1 and NPI dyes, impedance time constants and recombination lifetimes, Nyquists of IMPS measurements of RK1 and NPI dyes, and calculated diffusion coefficient and length, external and internal quantum efficiencies of the RK1 dye-sensitized solar cell, and flow chart of experiments for the EIS measurements under dark conditions of the activated NPI dye (PDF)
