# Supplementary material for: Characterization of Photochromic Dye Solar Cells Using Small-Signal Perturbation Techniques
Source: ACS Appl Energy Mater. 2021 Aug 4;4(9):8941–52. doi: 10.1021/acsaem.1c01204 (PMC8488939; doi:10.1021/acsaem.1c01204)
Supplement: Supplementary file 1 — ae1c01204_si_001.pdf [file ae1c01204_si_001.pdf]

Supporting Information - Characterization of photochromic dye solar cells using small-signal perturbation techniques

Antonio J. Riquelme,[†] Valid Mwatati Mwalukuku,[‡] Patricia Sánchez-Fernández,[†] Johan Liotier,[‡] Renán Escalante,[†] Gerko Oskam,[†,¶] Renaud Demadrille,[*,‡] and Juan A. Anta[*,†]

†*Área de Química Física, Universidad Pablo de Olavide, E-41013, Seville, Spain*

‡*University Grenoble Alpes, CEA, CNRS, Interdisciplinary Research Institute of Grenoble (IRIG), Molecular Systems and nanoMaterials for Energy and Health (SyMMES), Grenoble, France*

¶*Department of Applied Physics, CINVESTAV-IPN, Mérida, Yucatán, 97310, México*

E-mail: renaud.demadrille@cea.fr; anta@upo.es

Supporting Information Available

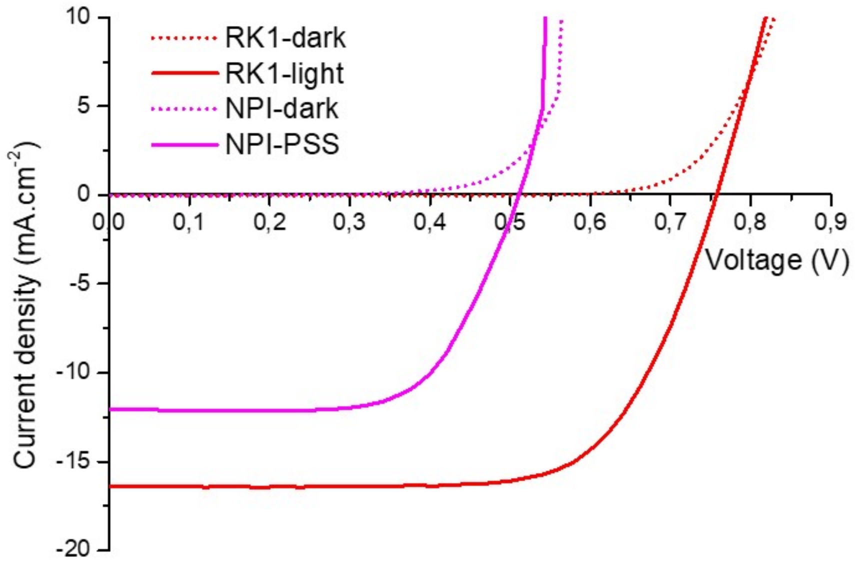

Figure S1: Current-voltage curves under dark conditions and under 1 sun illumination of the non photochromic RK1 DSSC compared to the photochromic NPI DSSC in the photo-stationary state.

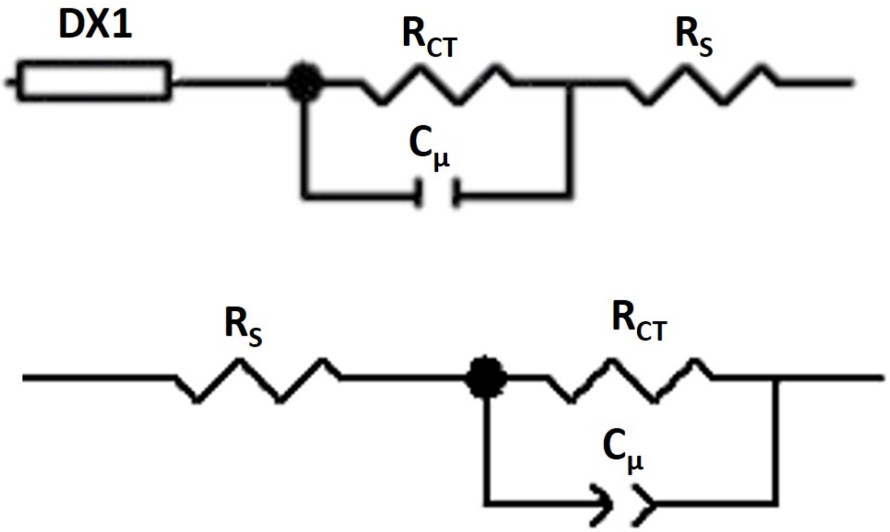

Figure S2: Typical equivalent circuit used to fit the EIS results of DSSCs (up). DX1 is an element that takes into account the diffusion-recombination transmission line. Simplified equivalent circuit used to fit the spectra obtained in this study (bottom).

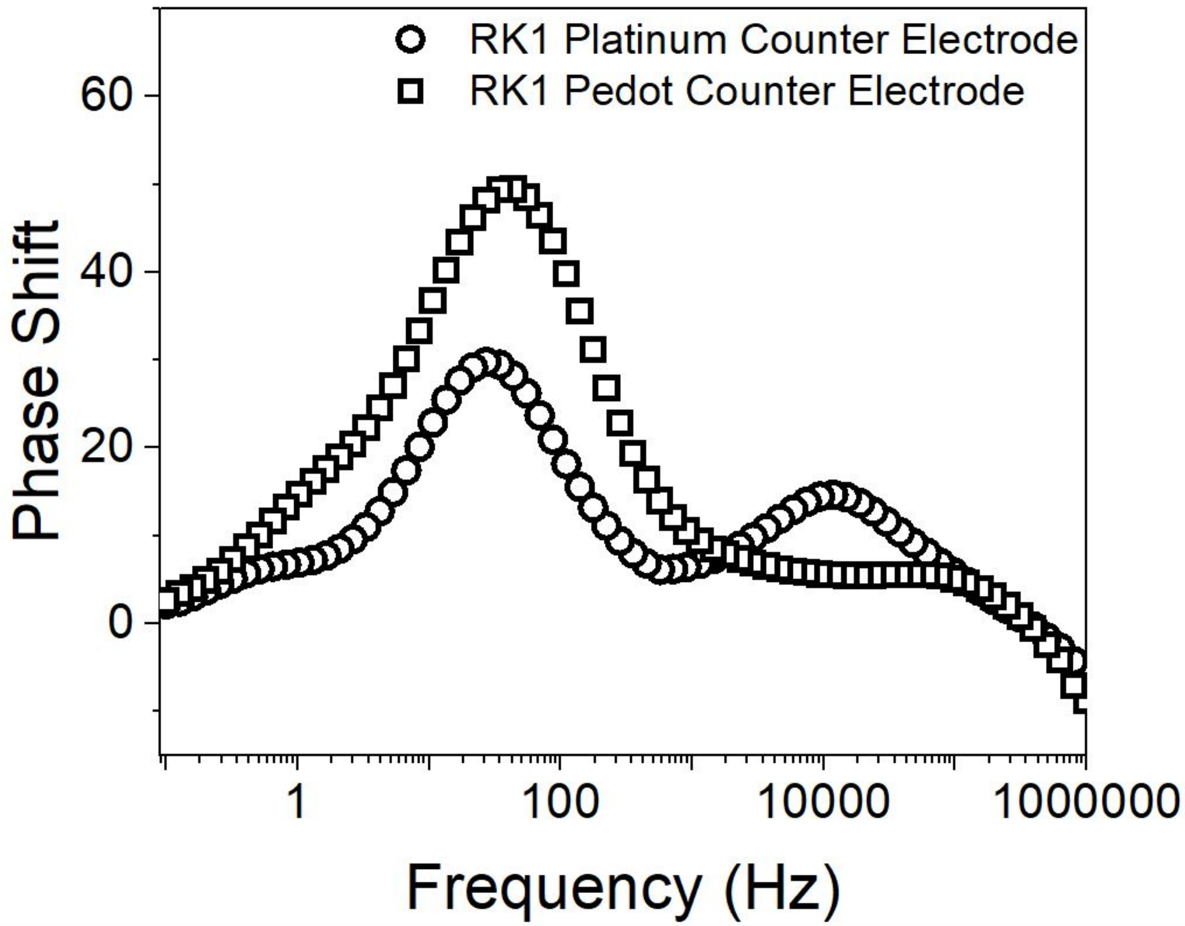

Figure S3: Bode plots from EIS for an RK1 dye solar cell at an applied voltage of 0.7 V, which is equal to the open circuit voltage under white illumination for a cell with a platinum (circles) and a PEDOT (squares) counter electrode.

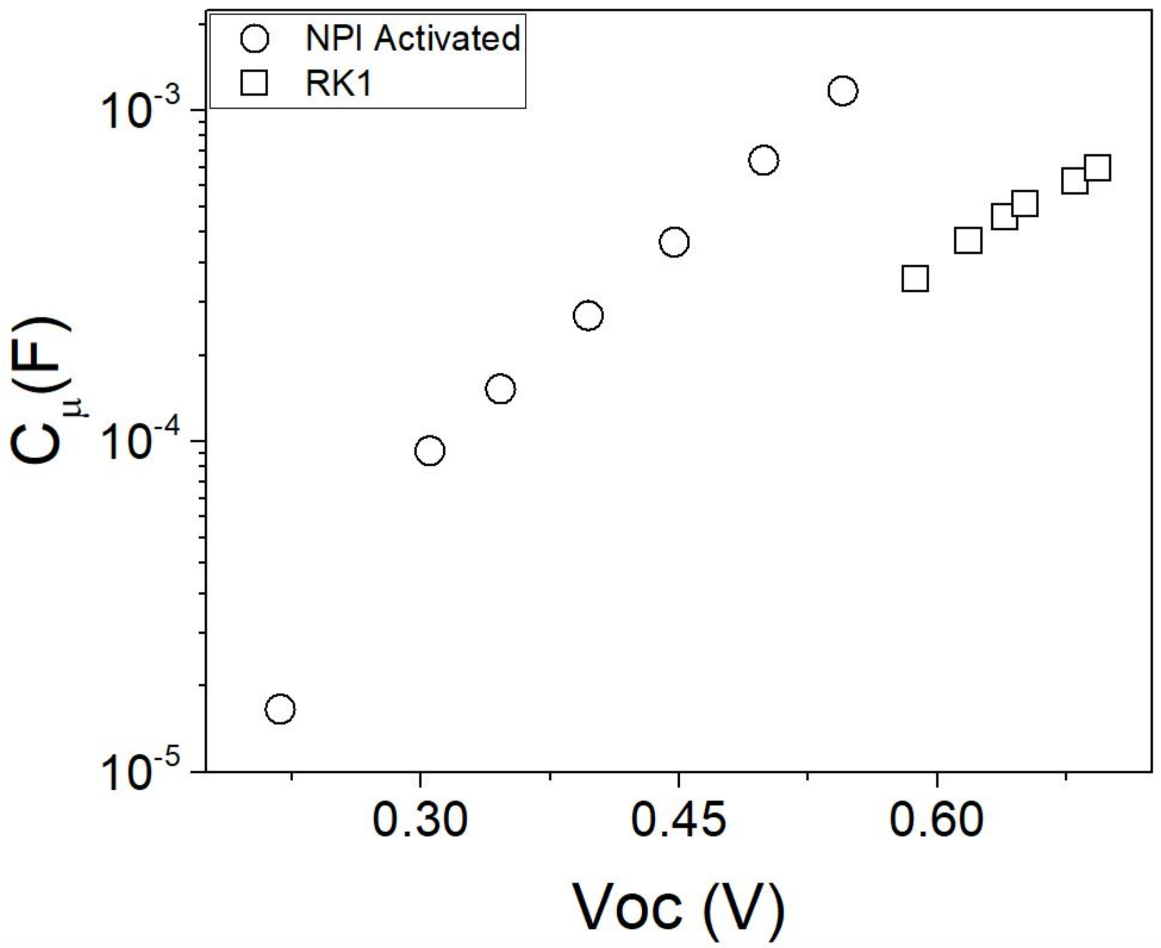

Figure S4: Chemical capacitances vs. applied voltage obtained from EIS with white light for RK1 and activated NP1 devices.

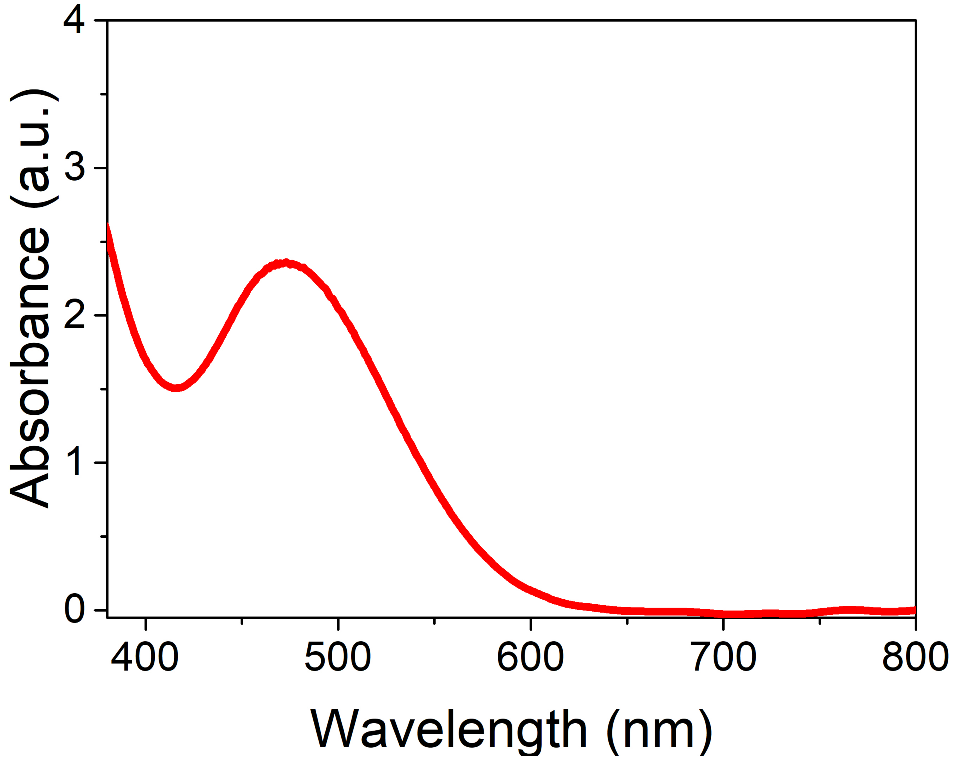

Figure S5: Absorbance spectrum for the 380 - 800 nm wavelength range of the RK1 dye on a 2 μm TiO_2 electrode.

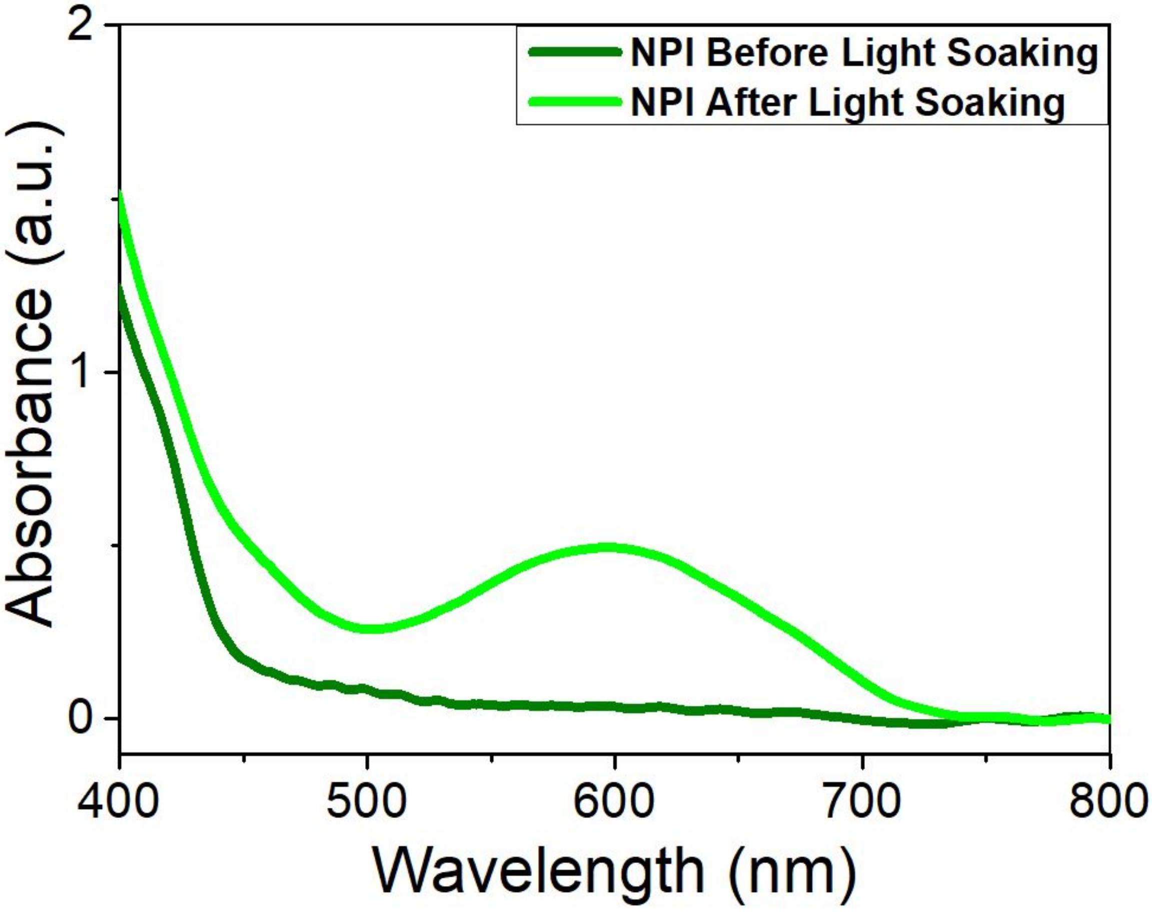

Figure S6: Absorbance spectrum for the 400 - 800 nm wavelength range of the photochromic NPI dye before and after light soaking on a 2 μm TiO_2 electrode.

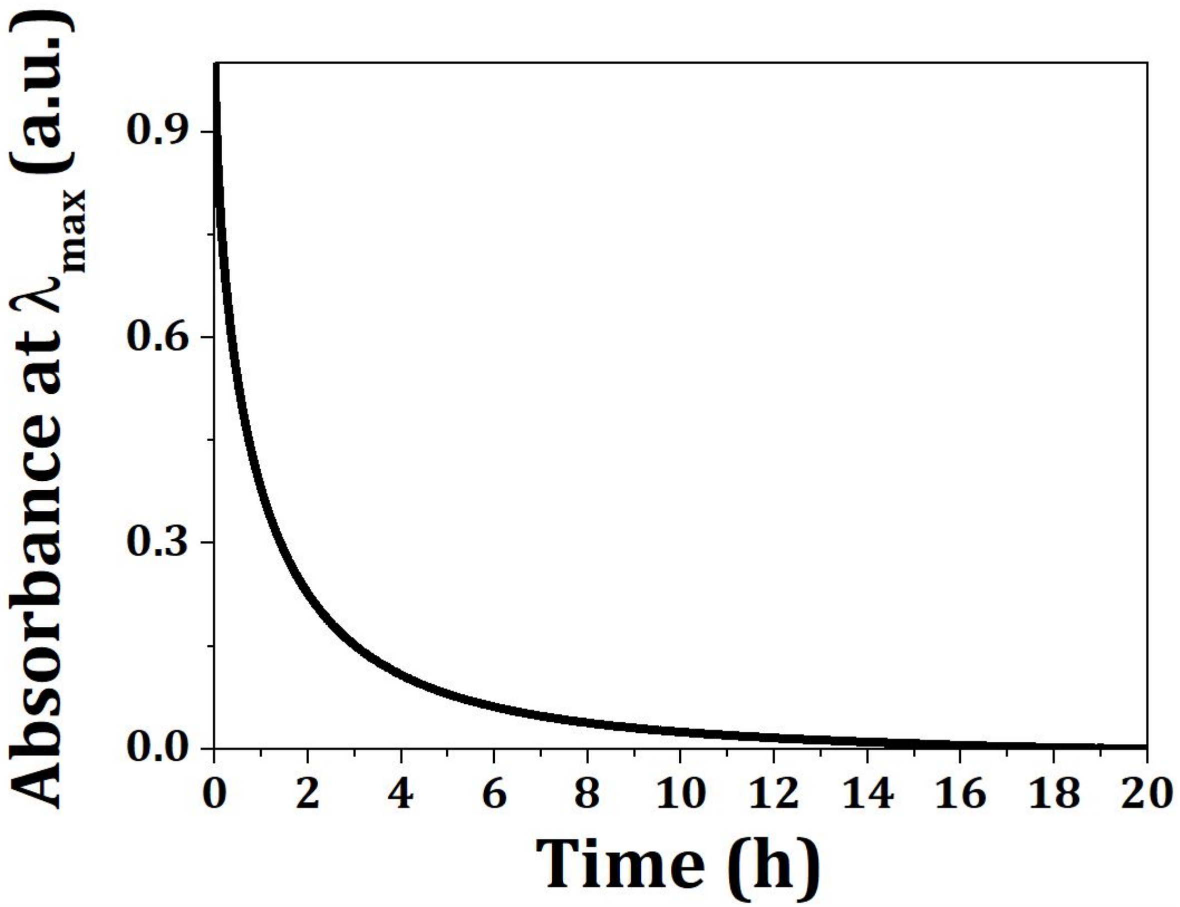

Figure S7: Deactivation kinetics of the photochromic NPI dye measured on a complete cell with a 13 μm TiO_2 electrode.

Table S1: α and β values obtained from fitting the EIS results of the RK1 solar cells to the parallel -RC- element and applying equations 1 and 2.

**Color Illumination**	α **value**	β **value**
Dark	0.35 ± 0.03	0.63 ± 0.01
White	0.28 ± 0.01	0.87 ± 0.02
Blue	0.24 ± 0.01	0.79 ± 0.07
Red	0.26 ± 0.04	0.75 ± 0.06

Table S2: α and β values obtained from fitting the EIS results of the NPI solar cells before and after light soaking to the parallel -RC- element and applying equations 1 and 2.

**Color Illumination**	α **value**	β **value**
Dark before light soaking	0.31 ± 0.01	0.55 ± 0.09
Dark after light soaking	0.27 ± 0.04	0.50 ± 0.06
White before light soaking	0.28 ± 0.01	0.80 ± 0.10
White after light soaking	0.27 ± 0.06	0.77 ± 0.02
Blue before light soaking	0.30 ± 0.04	0.83 ± 0.09
Blue after light soaking	0.29 ± 0.04	0.85 ± 0.07
Red before light soaking	0.31 ± 0.05	0.77 ± 0.04
Red after light soaking	0.28 ± 0.04	0.75 ± 0.08

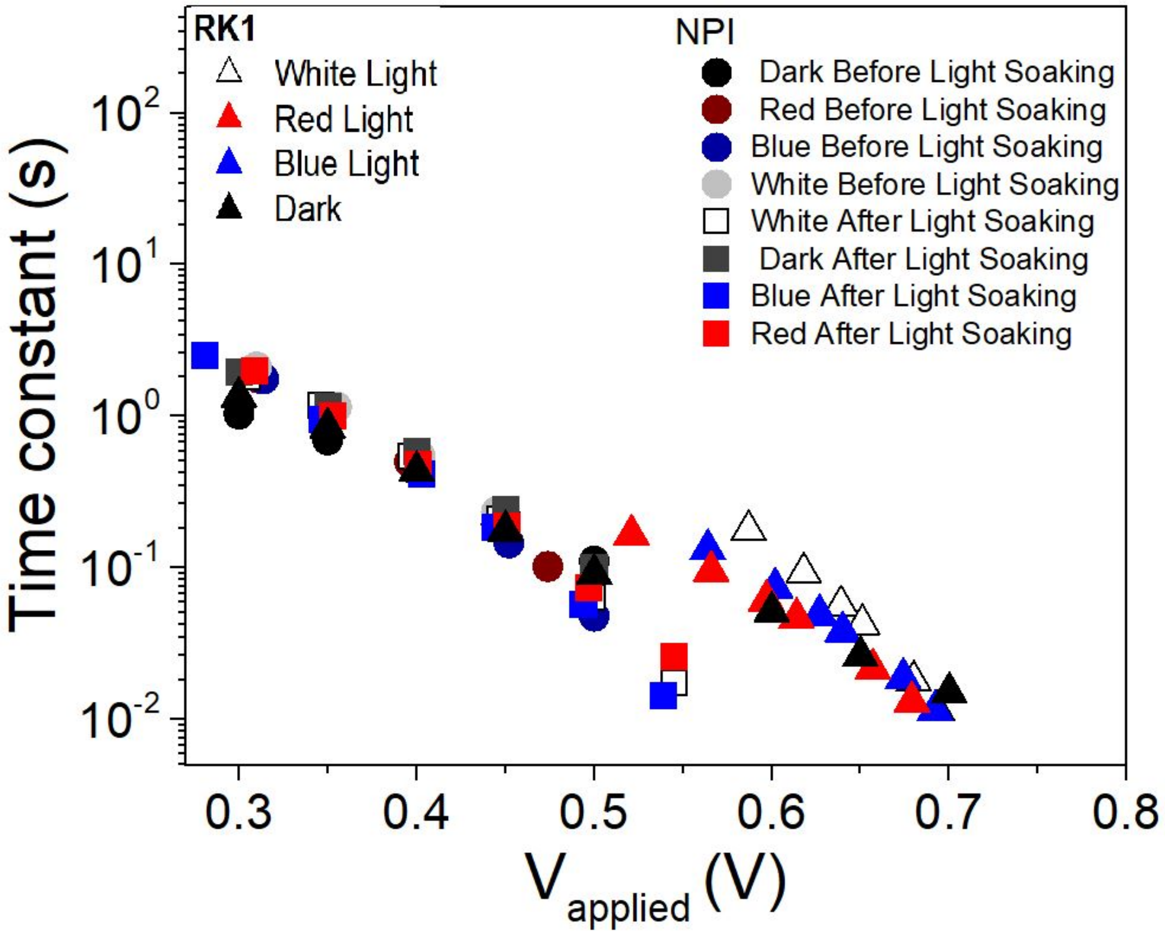

Figure S8: Time constants associated to the recombination feature of the EIS results under different sources of illumination for a RK1 and a NPI device extracted from the frequency of the main peak of the Bode plots in Figure 2.

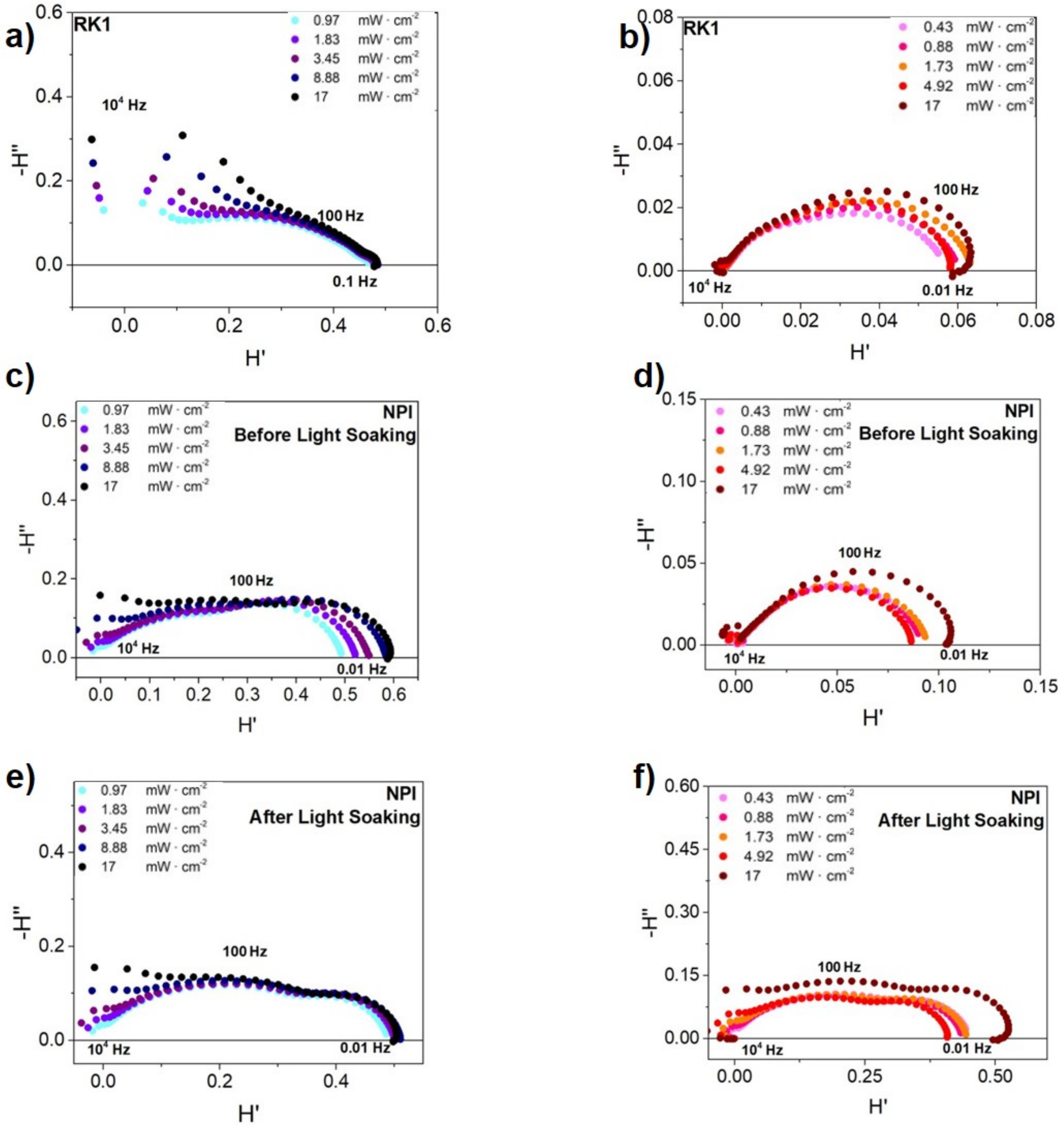

Figure S9: Nyquist plots of IMPS measurements for an RK1 dye sensitized solar cell (a and b) and a deactivated (c and d) and activated (e and f) NPI dye sensitized solar cell under different light intensities with blue (left) and red (right) illumination.

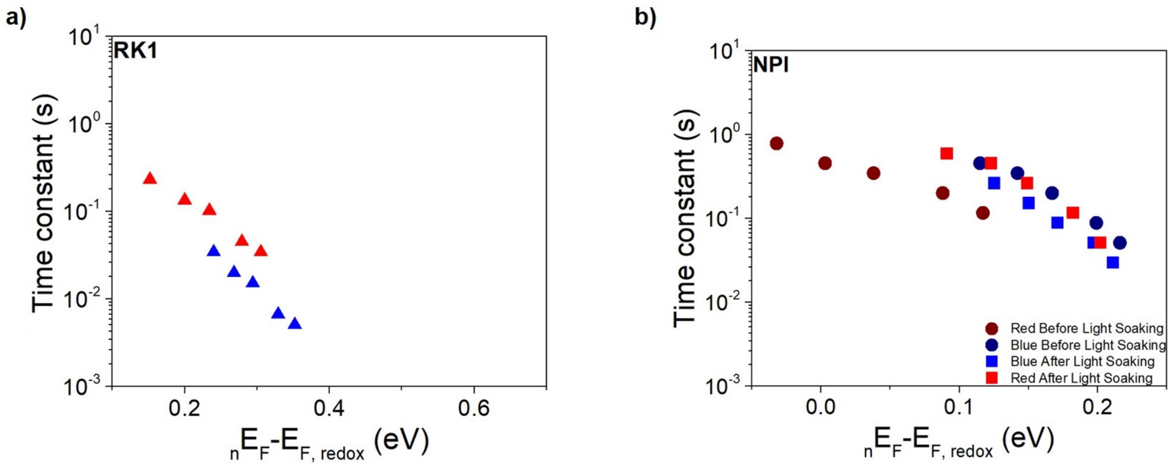

Figure S10: Time constants obtained from IMPS measurements for an RK1 (left) and a NPI (right) solar cell, with the corrected voltage to account for the difference between the trap occupancy between open-circuit and short-circuit conditions as described in the main text.

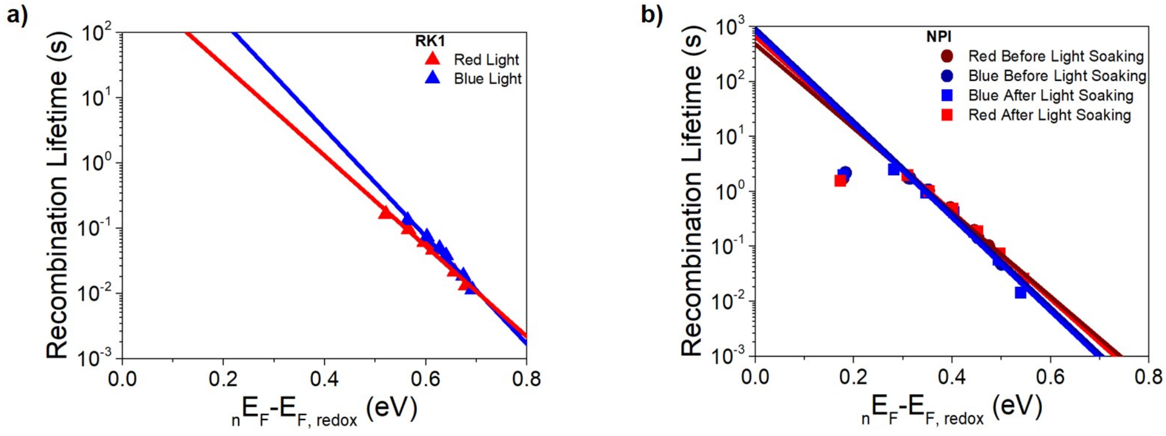

Figure S11: Recombination lifetime vs. corrected voltage obtained from the EIS measurements (points), and extrapolated to short circuit conditions (lines) of: a) an RK1 and b) a deactivated and activated NPI-based solar cell.

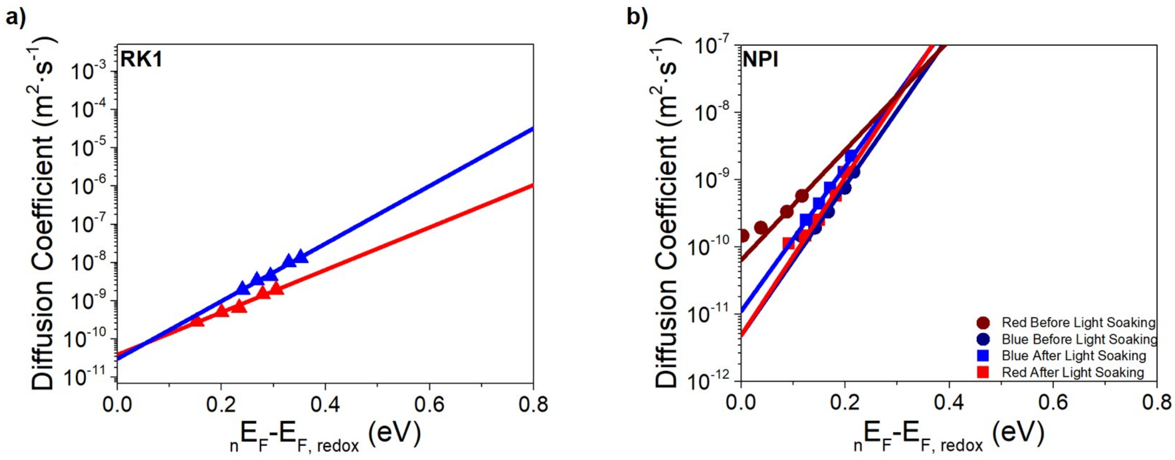

Figure S12: Diffusion coefficient vs. corrected voltage calculated from the IMPS time constants for an RK1 (left) and a deactivated and activated NPI (right) solar cell using equation 5, and extrapolated to short circuit conditions (trend lines).

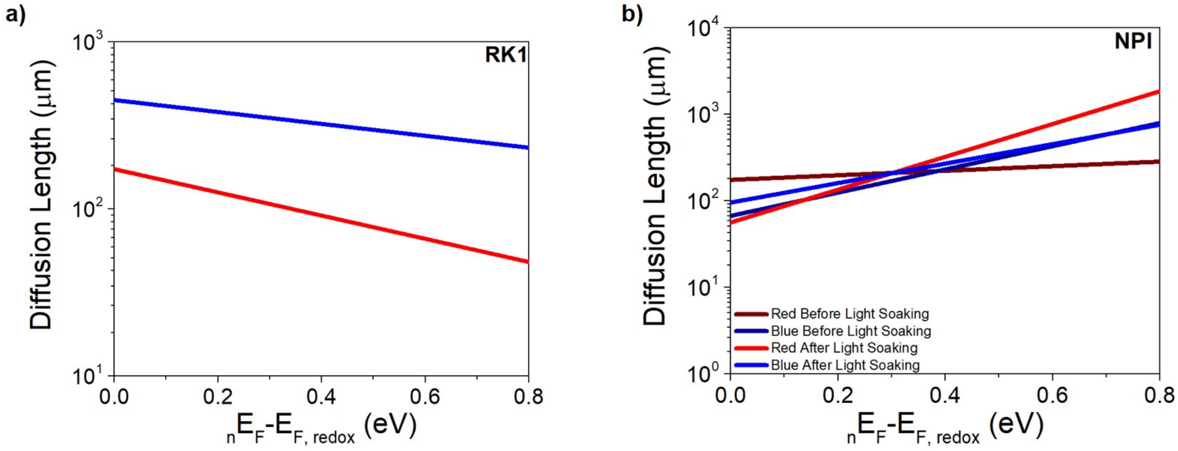

Figure S13: Diffusion length vs. corrected voltage for an RK1 (left) and a deactivated and activated NPI (right) solar cell calculated by multiplying the trendlines obtained in Figure S11 and Figure S12 according to Equation 4.

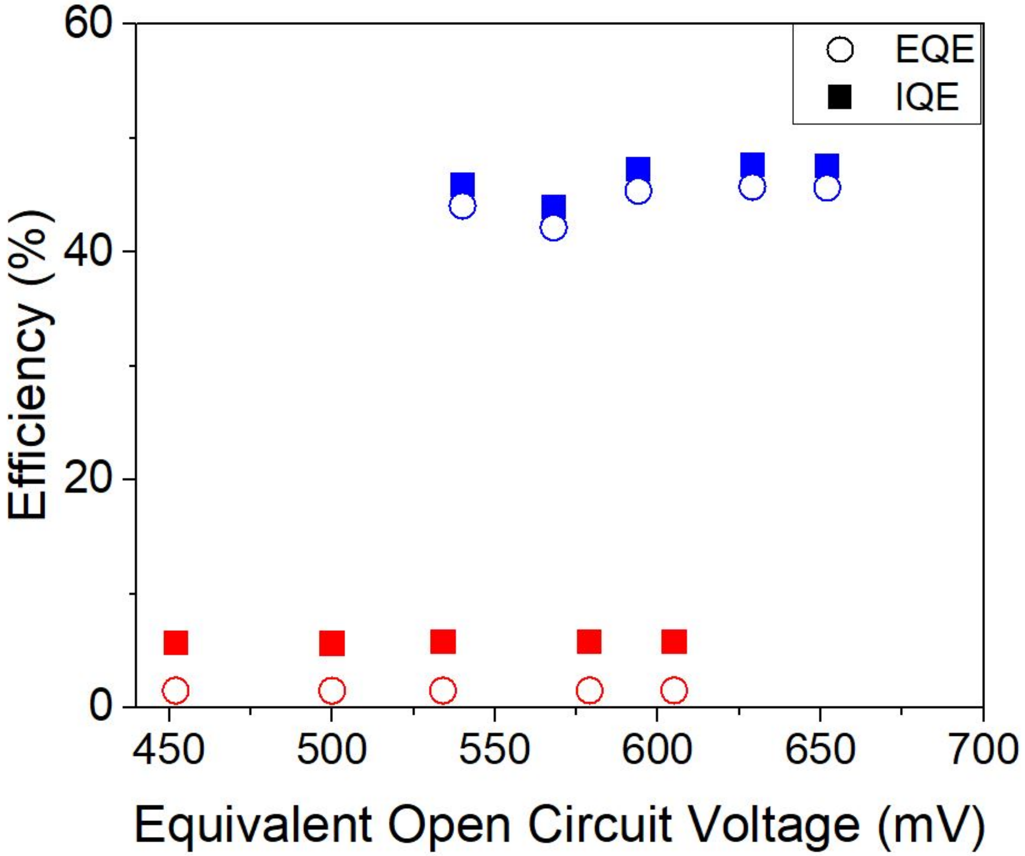

Figure S14: External (hollow symbols) and internal (solid symbols) quantum efficiency vs. equivalent open-circuit voltage obtained from IMPS measurements at short circuit under blue and red monochromatic illumination for an RK1 dye-sensitized solar cell.

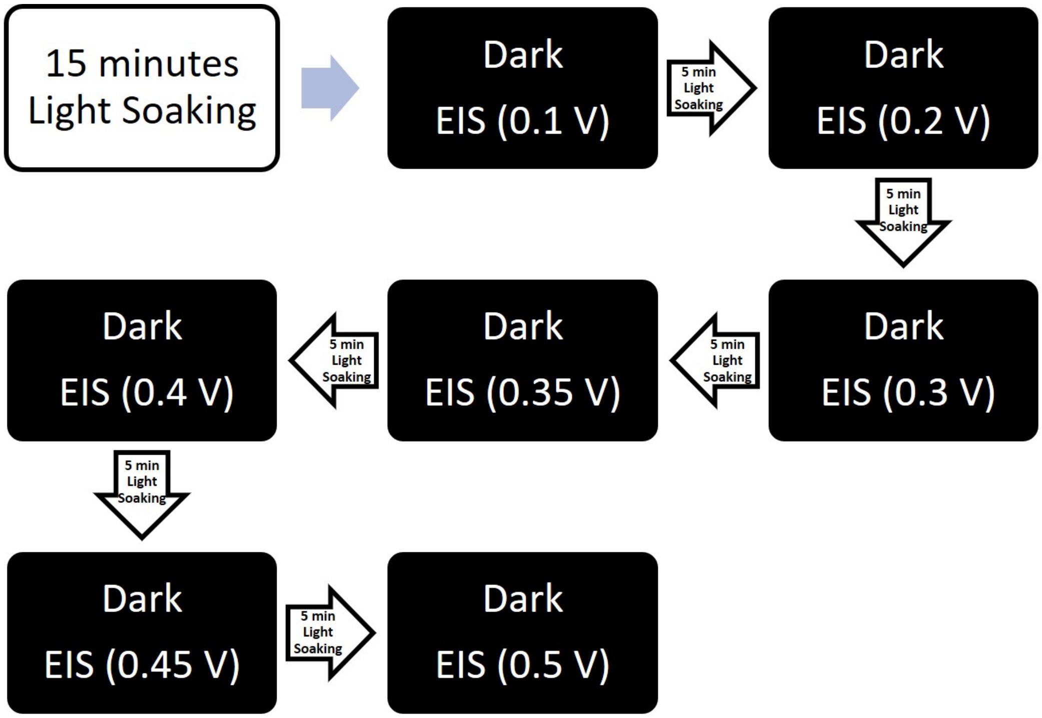

Figure S15: Flow chart of the order of experiments for the EIS measurements in the dark after light soaking in order to ensure a similar degree of activation for all the measurements.
